# Graphene-Based Composites for Thermoelectric Applications at Room Temperature

**DOI:** 10.3390/ma16237262

**Published:** 2023-11-21

**Authors:** Sonya Harizanova, Vassil Vulchev, Radostina Stoyanova

**Affiliations:** 1 Institute of General and Inorganic Chemistry, Bulgarian Academy of Sciences, 1113 Sofia, Bulgaria; 2 Faculty of Physics, University of Sofia, 1164 Sofia, Bulgaria; vdv@phys.uni-sofia.bg

**Keywords:** thermoelectric oxides, expanded graphite, graphene oxide, layered oxides, zinc oxide, multiphase composites

## Abstract

The thermoelectric materials that operate at room temperature represent a scientific challenge in finding chemical compositions with three optimized, independent parameters, namely electrical and thermal conductivity and the Seebeck coefficient. Here, we explore the concept of the formation of hybrid composites between carbon-based materials and oxides, with the aim of modifying their thermoelectric performance at room temperature. Two types of commercially available graphene-based materials are selected: N-containing reduced graphene oxide (NrGO) and expanded graphite (ExGr). Although the NrGO displays the lowest thermal conductivity at room temperature, the ExGr is characterized by the lowest electrical resistivity and a negative Seebeck coefficient. As oxides, we choose two perspective thermoelectric materials: p-type Ca_3_Co_4_O_9_ and n-type Zn_0.995_Al_0.005_O. The hybrid composites were prepared by mechanical milling, followed by a pelleting. The thermoelectric efficiency was evaluated on the basis of its measured electrical resistivity, Seebeck coefficient and thermal conductivity at room temperature. It was found that that 2 wt.% of ExGr or NrGO leads to an enhancement of the thermoelectric activity of Ca_3_Co_4_O_9_, while, for Zn_0.995_Al_0.005_O, the amount of ExGr varies between 5 and 20 wt.%. The effect of the composites’ morphology on the thermoelectric properties is discussed on the basis of SEM/EDS experiments.

## 1. Introduction

Oxide materials have been attracting attention due to their possible thermoelectric applications, since they exhibit a high Seebeck coefficient and thermal stability, together with low toxicity [1]. However, their relatively low electrical conductivity and high thermal conductivity cause obstacles for oxide commercialization as a thermoelectric material [1,2]. In general, the performance of thermoelectric materials is quantified by the dimensionless figure of merit ZT = S^2^T/(ρλ), where S is the Seebeck coefficient, T is the absolute temperature, ρ is the electrical resistivity, and λ is the thermal conductivity [3,4]. Based on these relations, high thermoelectric activity could be reached when oxides simultaneously have high electrical conductivity and low thermal conductivity [3,4]. In this respect, the most intriguing thermoelectric oxides comprise Co-based, strongly correlated systems (perovskites, LaCoO_3_, layered oxides, NaCo_2_O_4_ and RBaCo_2_O_5+x_, misfit layered oxides, Ca_4_Co_3_O_9_), a Ti-based Ruddlesden–Popper phase (SrTiO_3_), ZnO, etc. [5,6]. Given the diversity and flexibility of the crystal structures, the state-of-the-art approach for improving the thermoelectric performance of oxides is through selective metal substitution [5]. The metal substituents affect both the electronic structure and vibrational properties of oxides, thus contributing to augmented thermoelectric activity [5]. As each substituent only has an effect on one parameter (i.e., electrical or thermal conductivities), the use of more than one substituent enables the optimization of the several independent parameters and can help to achieve the precise control of thermoelectric properties [7,8]. Despite the effectiveness of this approach, the preparation of multiple substituted oxides is usually a complex and expensive process.

As an alternative to single and multiple substitution, the approach including the formation of composites between several distinct phases is becoming more attractive [9,10,11,12]. In multiphase composites, interfacial and texturized effects (the grain boundaries, confined particle sizes, oriented growth, etc.) give rise to the enhanced phonon-scattering and charge carrier mobility, which, in turn, lead to a better thermoelectric activity [13,14,15,16]. Two groups of multiphase composites need to be highlighted: multicomponent inorganic or organic composites and hybrid organic–inorganic composites [17]. The main feature of all organic or inorganic composites is their enhanced thermoelectric performance due to the combination of components with high electrical and low thermal conductivities [10,11,13,16]. However, the main disadvantage of the organic composites is their low Seebeck coefficients, while, for inorganic composites, their electrical conductivity is still far from the desired values. These disadvantages can be overcome by the formation of hybrid composites between inorganic and organic components, where the thermoelectric performance is dictated by the low thermal conductivity of the organic component and high Seebeck coefficient of the inorganic component [18]. In this respect, graphene has emerged as a competitive component thanks to its unique electrical conductivity and flexibility [19,20]. Unfortunately, the high thermal conductivity and low Seebeck coefficient restrict the graphene’s merit in the range of 10^−4^ [15,16]. Given the difficulties in the synthesis of pure graphene, reduced graphene oxide (rGO) has recently become the subject of intensive studies due to its cheaper and easier means of synthesis [9,21]. Based on DFT calculations, it has been shown that the thermal conductivity can be effectively reduced by the attachment of oxygen atoms to the graphene layers (i.e., the formation of graphene oxide) [22,23]. Contrary to graphene, graphene oxide exhibits lower electrical conductivity, which leads to a worsening of its thermoelectric activity [19,24]. The enhancement of electrical conductivity is achieved through a mild reduction in graphene oxide (i.e., rGO) [20,25]. Thus, rGO has recently become a main subject of study due to its thermoelectrical applications [9,26]. It has been reported that rGO can also be used as an additive to oxides in order to control their thermoelectric performance [27,28]. In addition to graphene and rGO, expanded graphite (ExGr) has also recently attracted research interest [29,30]. ExGr has been utilized as a filler to multifunctional cement composites [31,32].

Despite the variety of studies of hybrid composites, there are still few investigations on thermoelectric materials that are able to operate at room temperature [33,34]. At present, the main room-temperature thermoelectric materials rely on BiTe-, S-, and Se-based alloys, which are highly toxic [29,30]. Contrary to the alloy-based materials, environmentally benign oxides performed worse at room temperature [35,36]. The question is whether the room-temperature thermoelectric efficiency could be improved by the formation of hybrid composites.

Here, we explore the concept of the formation of hybrid composites between carbon-based materials and oxides, with the aim of modifying their thermoelectric performance at room temperature. Three types of commercially available graphene-based materials are selected: reduced graphene oxide (rGO), N-containing reduced graphene oxide (NrGO) and expanded graphite (ExGr). As oxides, we chose *p*-type Ca_3_Co_4_O_9_ and *n*-type Zn_0.995_Al_0.005_O. These oxides were selected due to their ability to modify the electrical and thermal properties through the regulation of the interface and boundary grains [37,38]. In addition, the thermal conductivity of oxides displays size-dependent effects, which allows for the suppression of thermal conductivity through effective phonon scattering [39]. Thus, we intend to modify the thermoelectric performance of these oxides at room temperature through the formation of composites with graphene-based materials. The hybrid composites were prepared by mechanical milling. The thermoelectric efficiency was evaluated on the basis of the measured electrical resistivity, Seebeck coefficient and thermal conductivity at room temperature. Based on SEM/EDS analysis, the effect of the composites’ morphology on the thermoelectric activity is discussed. The study of thermoelectric parameters will provide the necessary information on the applicability of the studied materials to the direct conversion of thermal energy into electrical energy and the temperature range in which the materials are most promising.

## 2. Materials and Methods

**Pristine oxides**, **carbon materials and synthetic procedures**. We used Ca(NO_3_)_2_.4H_2_O (Sigma Aldrich, St. Louis, MO, USA), Co(NO_3_)_2_.6H_2_O, Zn(NO_3_)_2_.6H_2_O (Sigma Aldrich, St. Louis, MO, USA), Al(NO_3_)_3_.9H_2_O (Sigma Aldrich, St. Louis, MO, USA), citric acid (p.a. Chem-Solutions, GmbH, Dimitrovgrad, Bulgaria) and NH_4_HCO_3_ (p.a. Chem-Solutions, GmbH, Dimitrovgrad, Bulgaria) as reagents. Layered Ca_3_Co_4_O_9_ was obtained by a Pechini-type reaction, as described elsewhere [10]. The solution containing Ca(NO_3_)_2_.4H_2_O, Co(NO_3_)_2_.6H_2_O, citric acid and ethylene glycol (at a ratio of Ca:Co:CA:EG = 1:1:10:40) was heated at 90 °C until drying and polyesterification; then, the solid residue was heated at 400 °C for 3 h. Aluminum-doped zinc oxide (Zn_0.995_Al_0.005_O with wurtzite-type structure) was prepared by co-precipitation from an aqueous solution of zinc and aluminum nitrates with NH_4_HCO_3_; the details are provided elsewhere [40]. The precursors were tableted and annealed at a specific temperature depending on the oxide composition: Ca_3_Co_4_O_9_ was annealed at 800 °C for 20 h in an oxygen atmosphere, while Zn_0.995_Al_0.005_O was obtained at 750 °C for 10 h in air. The reduced graphene oxide (rGO), N-doped reduced graphene oxide (NrGO) and expanded graphite (EXG 98 20/20 µm) are commercial products provided by Graphit Kropfmühl GmbH (Hauzenberg, Germany).

**Preparation of composites**. The composites between oxides (Ca_3_Co_4_O_9_ or Zn_0.995_Al_0.005_O) and carbon additives (rGO, NrGO or ExCr) were fabricated through the mechanical mixing of the given oxide with a carbon material at a weight ratio of 98:2%, 95:5%, and 80:20%. The mixtures were pelleted and thermally treated at 200 °C for 5 h in argon atmosphere. For the sake of convenience, the composites will be denoted as follows: Ca_98_NrGO_2_, Ca_95_NrGO_5_ and Ca_80_NrGO_20_; Ca_98_ExGr_2_, Ca_95_ExGr_5_ and Ca_80_ExGr_20_; Zn_98_ExGr_2_, Zn_95_ExGr_5_ and Zn_80_ExGr_20_; Zn_98_NrGO_2_, Zn_95_NrGO_5_ and Zn_80_NrGO_20_.

**Characterization**. A structural analysis of samples was carried out using a powder X-ray diffractometer (Bruker Advance D8, Karlsruhe, Germany) equipped with LynxEye detector (CuKα). The morphology was analysed through scanning electron microscopy (SEM). SEM images of pellets along the surface and cross-section were monitored by a JEOL JSM 6390 microscope equipped with an EDS analyzer (Oxford INCA Energy 350) in a regime of secondary electron images (SEIs).

For the thermoelectrical characterization, square pellets with a dimension of 9 mm and thickness of about 1–2 mm of were fabricated. The pellet porosity was evaluated by a comparison of the pellet density (determined by the Archimedes method) with the theoretical density of Ca_3_Co_4_O_9_ and Zn_0.995_Al_0.005_O. The results indicate that the pellet porosity varied at around 25%, irrespective of its phase composition. This allows for us to correctly compare the thermal properties of composites.

The electrical resistivity was determined by MMR’s Variable Temperature Hall System (K2500-5SLP-SP) within the framework of the Van der Pauw method. Thermal conductivity was calculated using a C-Therm TCi Thermal Conductivity Analyzer (MTPS). The Seebeck coefficient of the samples was measured at room temperature using the in-house setup. The holder comprises two solid plates with thermocouples. One of the plates contains a gradient heater. The measured sample was inserted between the solid plates (Figure S1). Then, the Seebeck voltage and temperature difference were recorded upon reaching the stationary condition. All parameters (i.e., electrical resistivity, thermal conductivity and Seebeck coefficient) were measured at 298 K.

## 3. Results

### 3.1. Thermoelectric Properties of rGO, NrGO and ExGr

The thermoelectrical properties of expanded graphite, rGO, and NrGO are compared in Table 1. The comparison shows that the expanded graphite exhibits the lowest electrical resisitivity (i.e., around 9.6 × 10^−4^ Ω.cm) and Seebeck coefficient (i.e., −26.1 μ/K), resulting in the highest power factor (PF) even at room temperature (i.e., 71 μW/(m.K^2^) at 20 °C). The magnitude of the PF is close to that determined for commercial-graphite-produced composite films (i.e., 87 μW/(m.K^2^) at 25 °C and 94 μW/(m.K^2^) at 150 °C) [29]. It is worth mentioning that the sign of the Seebeck coefficient is negative. Despite the relatively good magnitude of the PF, the high thermal conductivity of ExGr gives rise to a lower figure of merit. In comparison with the expanded graphite, both rGO and NrGO are characterized by their extremely low thermal conductivities (i.e., more than two orders lower than that of ExGr), but their figures of merit remain small, making them unsuitable for practical application. The close inspection of the data for rGO and NrGO indicates that both the power factor and figure of merit reach higher magnitudes for NrGO. It is of importance that the figure of merit for NrGO varies in the range of 10^−3^, which is slightly higher from the previously reported data (i.e., about 10^−4^) [19,20]. As far as we known, these are the first data on the thermoelectric activity of reduced graphene oxide where the O atoms are replaced by N. This motivated us to use only NrGO as a component in multiphase composites.

### 3.2. Multiphase Composites between Oxides and Carbon Additives

**Figure 1:**
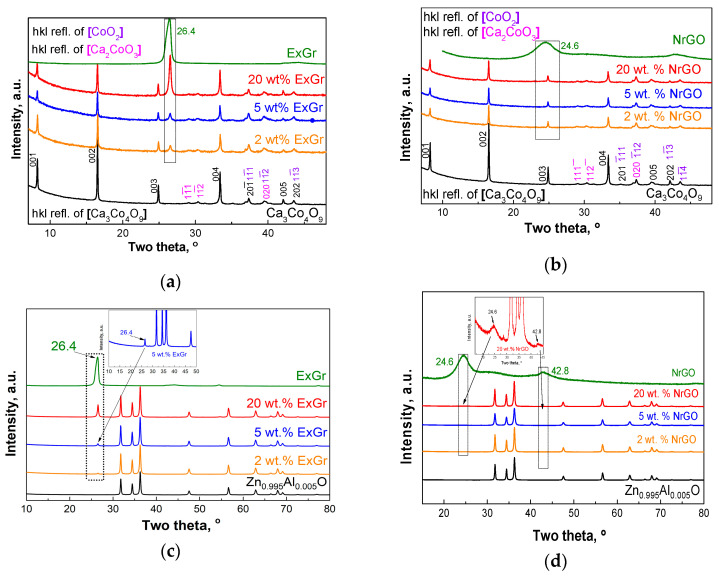
XRD patterns of composites of Ca_3_Co_4_O_9_ (**a**,**b**) and Zn_0.995_Al_0.05_O (**c**,**d**) with ExGr (**a**,**c**) and NrGO (**b**,**d**).

The ball-milling of oxides with carbon additives yields composites in which every individual component (oxide, NrGO, or ExGr) retains its structure. Figure 1 compares the XRD patterns of oxide–carbon composites. The indexation of XRD patterns shows the appearance of diffraction peaks due to Ca_3_Co_4_O_9_ and expanded graphite phases. In the case of NrGO, the diffraction peak (at around 24.6°) is too broad, thus preventing its observation in the XRD patterns of composites. The lattice parameters for Ca_3_Co_4_O_9_ and Zn_0.995_Al_0.005_O components are not changed during the composites’ formation and correspond to the previously reported parameters [10,37]: a = 4.8249 Å; b_1_ = 4.5687 Å; c = 10.862 Å; b_2_ = 2.8096 Å; β = 98.4° for Ca_3_Co_4_O_9_ and a = 3.2504 Å; c = 5.2062 Å for Zn_0.995_Al_0.005_O.

### 3.3. Effect of NrGO and ExGr on the Thermoelectric Properties of Ca_3_Co_4_O_9_

The formation of multiphase composites enables to compare the effect of carbon components on the thermoelectric properties of Ca_3_Co_4_O_9_. The ExGr has excellent electrical conductivity, which is about two orders higher than that of the oxide. In contrast, the electrical conductivity of the oxide slightly exceeds that of the NrGO (Table 1). Although the Seebeck coefficient has a positive sign for the oxide, the negative Seebeck coefficient is observed for ExGr and NrGO (Table 1). This reveals that different types of charge carriers are responsible for the electrical properties: for the oxide, the p-type is responsible, while for the ExGr and NrGO, the n-type is responsible. In terms of magnitude, the oxide Seebeck coefficient is the highest. Because of the excellent electrical conductivity, the power factor of the ExGr reaches the highest magnitude irrespective of its low Seebeck coefficient (Table 1). The thermal conductivity increases following the order NrGO < Ca_3_Co_4_O_9_ < ExGr. Combining all parameters into the figure of merit, it appears that ExGr and Ca_3_Co_4_O_9_ have comparable thermoelectric activities (0.025 and 0.013 at 20 °C, respectively), which outperform those of NrGO.

**Figure 2:**
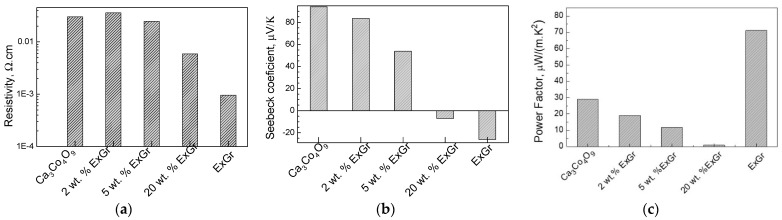
Electrical resistivity (**a**), Seebeck coefficient (**b**), and power factor (**c**) of the composites between Ca_3_Co_4_O_9_ and ExGr.

The addition of ExGr in amounts of up to 5 wt.% to Ca_3_Co_4_O_9_ only slightly affects the electrical resistivity, while the next amount of ExGr significantly reduces the electrical resistivity (Figure 2a). Along with the observed trend in changes in the electrical resistivity, the Seebeck coefficient progressively decreases, but its sign remains positive up to 5 wt.% of ExGr (Figure 2b). At 20 wt.% of ExGr, the sign of Seebeck is converted from positive to negative, reaching the value of the ExGr component. The simultaneous changes in the electrical resistivity and Seebeck coefficient could be explained in terms of the opposite charge carrier types observed for the individual components Ca_3_Co_4_O_9_ and ExGr (p- and n-type, respectively), as well as the average values of the composites’ electrical resistivity, measured between Ca_3_Co_4_O_9_ and ExGr components. Considering the electrical resistivity and Seebeck coefficient, the power factor of the Ca_3_Co_4_O_9_-ExGr composites is lower than that of the individual Ca_3_Co_4_O_9_ and ExGr components (Figure 2c).

**Figure 3:**
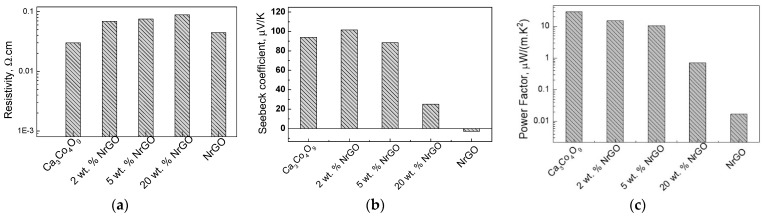
Electrical resistivity (**a**), Seebeck coefficient (**b**), and power factor (**c**) of the composites between Ca_3_Co_4_O_9_ and NrGO.

In comparison with the ExCr additives, the addition of NrGO leads to an increase in the electrical resistivity of Ca_3_Co_4_O_9_ (Figure 3a). It is interesting that the electrical resistivity of the Ca_3_Co_4_O_9_-NrGO composites is slightly higher than that of the individual Ca_3_Co_4_O_9_ and NrGO components. Up to 5 wt.% of NrGO, the Seebeck coefficient varies around the magnitude of the oxide component, followed by a drastic decrease after further increases in the NrGO content (Figure 3b). However, the sign of the Seebeck coefficient remains positive between 2 and 20 wt.% of NrGO, which is different to that of the ExGr-containing composites. The different trends in variation of the electrical resistivity and Seebeck coefficients of ExGr and NrGO-containing composites are, most probably, related to the specific manner of packing of the oxide particles using carbon additives: it appears that ExGr more strongly modifies the oxide in comparison with NrGO. (This will be discussed in the next section.) Irrespective of these different behaviors, the power factor of Ca_3_Co_4_O_9_ is almost unchanged for up to 5 wt.% of NrGO addition, as in the case of ExGr-containing composites (Figure 3c). All these data disclose that small amounts of ExGr and NrGO (up to 5 wt.%) slightly affect the power factor of the oxide.

**Figure 4:**
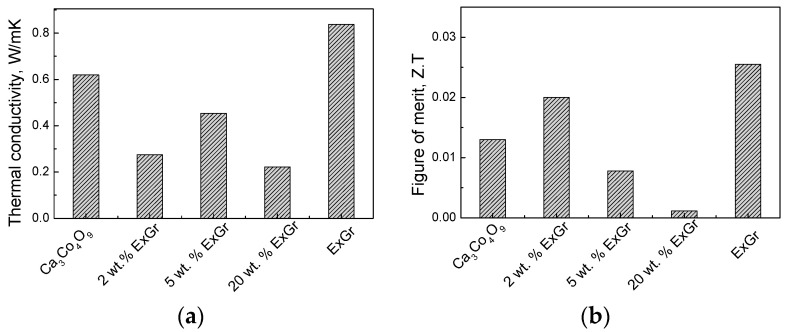

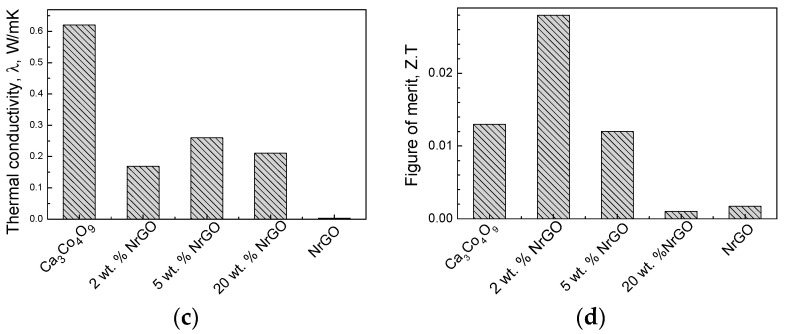
Thermal conductivity (**a**,**c**) and figure of merit (**b**,**d**) for the composites of Ca_3_Co_4_O_9_ with ExGr (**a**,**b**) and with NrGO (**c**,**d**).

The parameter that undergoes a strong change after the addition of carbon is the thermal conductivity (Figure 4). For the Ca_3_Co_4_O_9_-ExGr composites, the thermal conductivity strongly decreases, so they become smaller than the individual Ca_3_Co_4_O_9_ and ExGr components (Figure 4a). To rationalize this dependence, the contribution of the conductive carriers and phonon scattering to the overall thermal conductivity (λ) was taken into account. In the case when only one type of charge carrier contributes to the electron conductivity, the electrical thermal conductivity (λ_e_) is inversely proportional to the electrical resistivity (ρ) according to the Wiedemann–Franz law: λ_e_ = LT/ρ, where L is the Lorentz number (2.45 × 10^−8^ V^2^/K^2^) [7]. Taking into account the experimental data on the electrical resistivity of composites, the calculated conductive-carrier-induced thermal conductivity of Ca_3_Co_4_O_9_ increases with the ExGr content, as follows: 0.024, 0.021, 0.030, 0.125, and 0.764 W/(mK) for Ca_3_Co_4_O_9_; Ca_98_ExGr_2_, Ca_95_ExGr_5_, Ca_80_ExGr_20_, and ExGr, respectively. The comparison shows that the calculated electrical thermal conductivity is significantly lower than the experimentally measured values for the Ca_3_Co_4_O_9_ component (i.e., 0.62 W/(m.K)), signifying the leading role of phonon scattering in the overall thermal conductivity (about 96% of overall λ). Contrary to Ca_3_Co_4_O_9_, the overall thermal conductivity of ExGr (i.e., 0.838 W/(m.K)) is governed by conductive carriers. For the Ca_3_Co_4_O_9_-ExGr composites, phonon scattering remains a leading term in the overall thermal conductivity for up to 5 wt.% of ExGr (about 93% of the overall λ), while, at around 20 wt.% of ExGr, the role of conductive carriers dramatically increases, reaching 43% of the overall λ. Since the thermal conductivities of the composites are lower than those of the individual Ca_3_Co_4_O_9_ and ExGr components, it is possible to assume that phonon-boundary scattering is an important factor. It is now recognized that, through increasing the phonon scattering at grain boundaries, it is possibly effectively to reduce the thermal conductivity [13,41]. In other words, this is a future aim for the modification of the oxide interfaces through the addition of ExGr. The smaller thermal conductivity suggests an improvement in the figure of merit, with this improvement being best for Ca_98_ExGr_2_ (Figure 4b).

The NrGO has the same effect as the ExGr: the thermal conductivity of the Ca_3_Co_4_O_9_-NrGO composites decreases after the addition of small amounts of NrGO (i.e., 2 wt.%) (Figure 4c), but remains higher than that of the NrGO component. It is worth mentioning that the overall thermal conductivity of NrGO is also governed by the conductive carriers as in the case of ExGr: thus, the higher electrical resistivity of NrGO determines its smaller thermal conductivity compared to that of ExGr (Table 1). However, for the Ca_3_Co_4_O_9_-NrGO composites, the conductive-carrier-induced thermal conductivity makes a small contribution to the overall thermal conductivity (i.e., around 3–7% of λ) in the concentration range of 2–20 wt.%, a phenomenon that has already been observed for Ca_3_Co_4_O_9_-ExGr composites. Because of the lowest thermal conductivity of NrGO, the thermal conductivities of NrGO-containing composites are lower in magnitude in comparison with those for Ca_3_Co_4_O_9_-ExGr composites. This means that, irrespective of the stronger modification effect of ExGr on the oxide interface, the figure of merit of Ca_3_Co_4_O_9_-NrGO composites will be higher than that of Ca_3_Co_4_O_9_-ExGr composites. Thus, the best figure of merit is observed for the composite with 2 wt.% NrGO, which is higher than that of the composite containing 2 wt.% of ExGr (Figure 4d). It is of importance that even 2 wt.% of ExGr or NrGO is sufficient to improve the thermoelectric performance of the Ca_3_Co_4_O_9_ oxide.

### 3.4. Effect of NrGO and ExGr on the Thermoelectric Properties of Zn_0.995_Al_0.005_O

Although Ca_3_Co_4_O_9_ belongs to the p-type thermoelectric materials, the Al-doped ZnO is classified as n-type [42,43]. Zn_0.995_Al_0.005_O displays a large Seebeck coefficient with a negative sign (−590 μV/K), but the electrical resistivity is extremely high (Table 1). It is worth mentioning that Al doping is needed to improve the electrical properties of ZnO [37,38,39]. In this case, the electrical resistivity remains higher in comparison with other thermoelectric oxides (i.e., Ca_3_Co_4_O_9_). Thus, irrespective of the high magnitude of the Seebeck coefficient, the high electrical resistivity determines the small power factor for Zn_0.995_Al_0.005_O (i.e., about 10^−2^ μW/(m.K^2^), Table 1).

**Figure 5:**
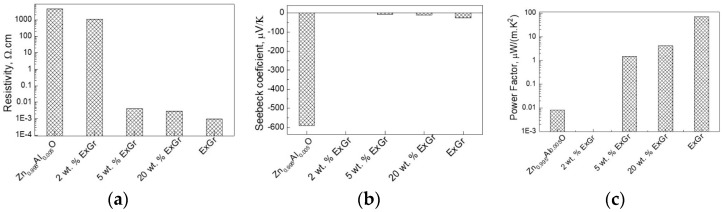

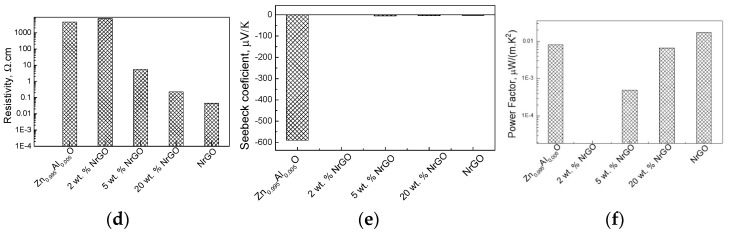
Electrical resistivity (**a**,**d**), Seebeck coefficient (**b**,**e**), and power factor (**c**,**f**) of the composites Zn_0.995_Al_0.005_O-ExGr (**top**) and Zn_0.995_Al_0.005_O-NrGO (**bottom**).

The addition of both ExGr and NrGO acts in the same manner. ExGr and NrGO yield a drastic decrease in the electrical resistivity of Zn_0.995_Al_0.005_O (Figure 5). The optimal content of carbon additives is between 5 and 20 at %: in this case, the electrical conductivities of the composites approach those of the individual ExGr component or NrGO. Because of the low electrical resistivity of ExGr, the Zn_80_ExGr_20_ composite outperforms the analogue, with 20 wt.% NrGO (i.e., an electrical resistivity of 2.9 × 10^−3^ Ω.cm for Zn_95_ExGr_5_ versus 2.3 × 10^−1^ Ω.cm for Zn_80_NrGO_20_). Along with the electrical resistivity, the Seebeck coefficient is also decreased, but the sign always remains negative. It is noticeable that even small amounts of carbon additives provoke a dramatic decrease in the Seebeck coefficient, a phenomenon that is not observed for the Ca_3_Co_4_O_9_-containing composites. Notwithstanding, due to the low electrical resistivity, the highest power factor is observed for the composites between Zn_0.995_Al_0.005_O and ExGr in amounts between 5 to 20 wt.%. It is of importance that the magnitude of the power factor of Zn_95_ExGr_5_ and Zn_80_ExGr_20_ ((i.e., around 2 μW/(m.K^2^)) is three orders higher than that of the individual Zn_0.995_Al_0.005_O component.

**Figure 6:**
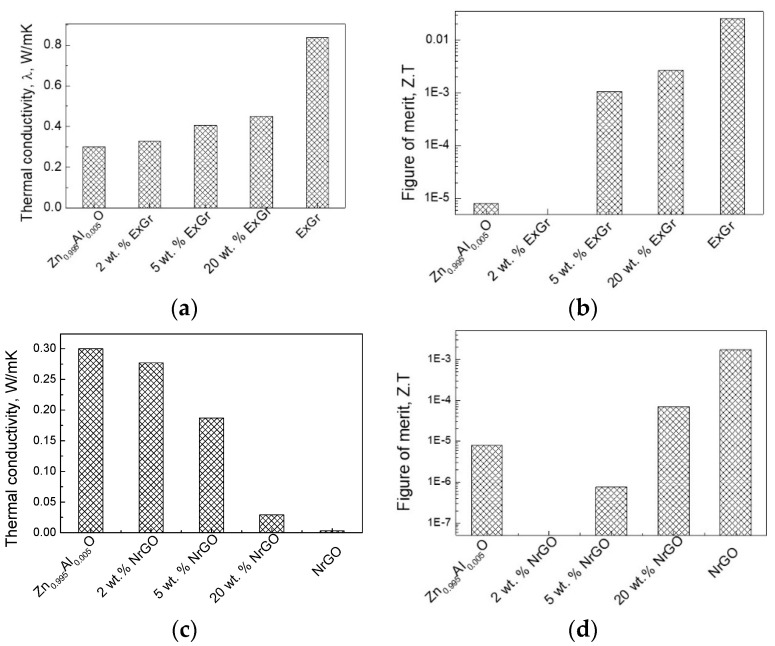
Thermal conductivity (**a**,**c**) and figure of merit (**b**,**d**) for the composites of Zn_0.995_Al_0.005_O with ExGr (**a**,**b**) and with NrGO (**c**,**d**).

In comparison with Ca_3_Co_4_O_9_, Zn_0.995_Al_0.005_O exhibits a lower thermal conductivity. However, as in the case of Ca_3_Co_4_O_9_, the thermal conductivity of Zn_0.995_Al_0.005_O is governed by phonon scattering (i.e., about 100% of overall λ). The addition of ExGr and NrGO to Zn_0.995_Al_0.005_O displays different effects on the thermal conductivity (Figure 6). After the addition of ExGr, the thermal conductivity of composites slightly increases in comparison with that of Zn_0.995_Al_0.005_O, but always remains lower than that of ExGr (Figure 6a). Irrespective of the higher thermal conductivity, and thanks to the better electrical conductivities, the Zn_0.995_Al_0.005_O-ExGr composites still exhibit a better figure of merit that that of Zn_0.995_Al_0.005_O. The NrGO causes a decrease in the thermal conductivity of Zn_0.995_Al_0.005_O, and at 20 wt.% of NrGO, the thermal conductivity of the composite approaches that of NrGO (Figure 6c). Thus, the composite with 20 wt.% of NrGO is characterized by a slight improvement in the figure of merit. The important result suggests that the thermoelectric activity of Zn_0.995_Al_0.005_O is improved thanks to the enhancement of the electrical conductivity of Zn_0.995_Al_0.005_O, especially after the addition of ExGr. Thus, the comparison evidences that ExGr plays a different role in the improvement in the thermoelectric properties of Zn_0.995_Al_0.005_O and Ca_3_Co_4_O_9_.

### 3.5. Composites Morphology

**Figure 7:**
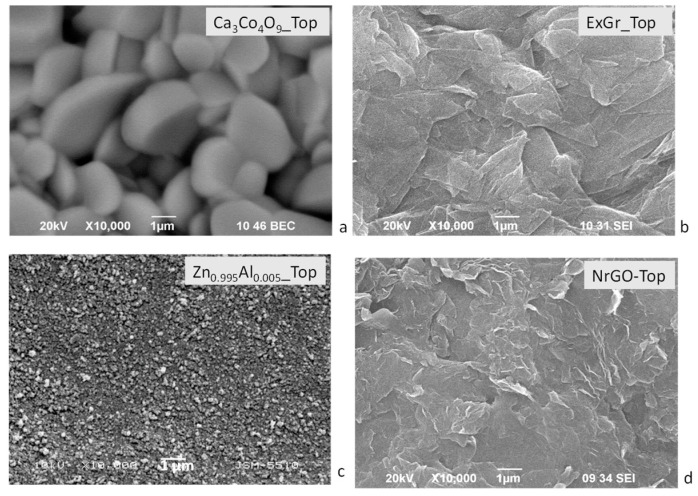
SEM images of the pellets containing Ca_3_Co_4_O_9_ (**a**), ExGr (**b**), Zn_0.995_Al_0.005_O (**c**) and NrGO (**d**). The top view of the pellet is shown for all compositions.

The dissimilar effects of ExGr and NrGO on the thermoelectric properties of oxides are closely related to the morphology of the composites. The morphologies of the individual oxide and carbon components are quite different (Figure 7). For Ca_3_Co_4_O_9_, well-shaped micrometric particles can be observed, while for Zn_0.995_Al_0.005_O, small, irregular nanometric particles occur. The different morphologies of Ca_3_Co_4_O_9_ and Zn_0.995_Al_0.005_O can be used to explain their thermal conductivities (Table 1): the smaller particles of Zn_0.995_Al_0.005_O ensure more effective routes for phonon scattering (such as grain boundaries), thus reducing the thermal conductivity. It has been reported that, for oxides with particle sizes below 100 nm, the thermal conductivity is reduced, which is of importance for their thermoelectric applications [44]. In comparison with oxides, the morphology of ExGr and NrGO consists of flake-like micrometric particles.

**Figure 8:**
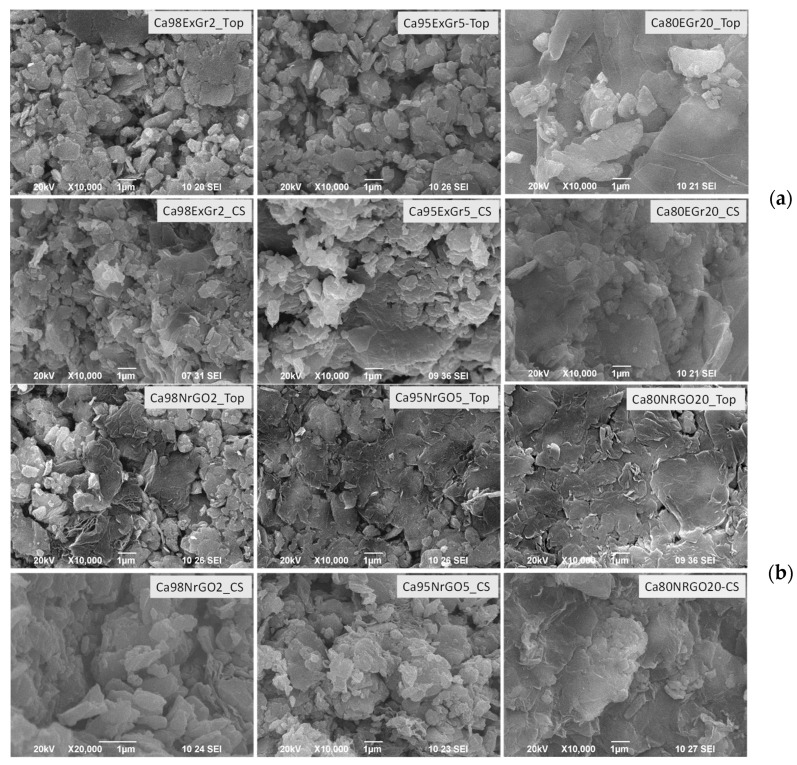
SEM images of the pellets (top and cross-sectional view) for the composites Ca_3_Co_4_O_9_-ExGr (**a**) and Ca_3_Co_4_O_9_-NrGO (**b**). The carbon additives increase from 2, 5 to 20 wt.% (from **left** to **right**).

In comparison with individual components, the morphology of the composites is changed. Since the thermoelectrical properties are measured using a square pellet, Figure 8 compares the SEM images of composites taken on the top and cross-section of the pellet. For the composites of Ca_3_Co_4_O_9_ with ExGr, the morphology on the pellet top consists of well-contacted micrometric and flake-like particles, with the relative part of the flake-like particles being increased according to the amount of ExGr. At the cross-sectional area of the pellet, the micrometric particles seem to dominate the flake-like ones. This picture is observed when ExGr is replaced with NrGO: on the pellet’s top, the flake-like particles are clearly seen, while at the pellet cross-section, micrometric particles mainly appear (Figure 8).

**Figure 9:**
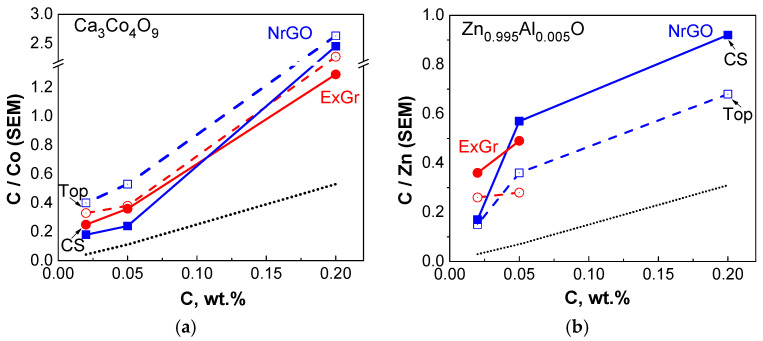
C-to-Co (**a**) and C-to-Zn ratio (**b**), determined from EDS-SEM of composites, as a function of the nominal carbon content in the given pellet. The open symbols correspond to the ratio determined on the top of the pellet, while the solid symbol reflects the ratio determined at the pellet’s cross-section. The calculated C-to-Co and C-to-Zn values from the nominal carbon content are indicated as dotted lines.

To check the SEM observations, an EDS analysis is undertaken (Figure S2). Figure 9 compares the C-to-Co ratio determined on the top and inside of the pellet. For accurate comparison, the element content for all samples was estimated from one and same area: x = 50.7 μm and y = 38.2 μm. In general, EDS accesses different depth profiles depending on the element’s nature, but these usually vary between 1 and 2 μm. (It should be noted that the pellet thickness is about 1–2 mm, which is about three orders higher than the EDS element’s profile depth). Irrespective of the lower accuracy in the EDS determination of the light C in comparison with the heavier Co, the C-to-Co ratio allows for the element distribution on the top of and inside the pellet to be monitored. The comparison clearly shows that the C-to-Co ratio is higher on the pellet’s top. This rule is obeyed for the oxide composites with ExGr and NrGO. The depth distribution of the carbon additives suggests the formation of a heterostructure along the pellet’s thickness. This will be the next source of phonon scattering, contributing to a reduction in the thermal conductivity of composites.

**Figure 10:**
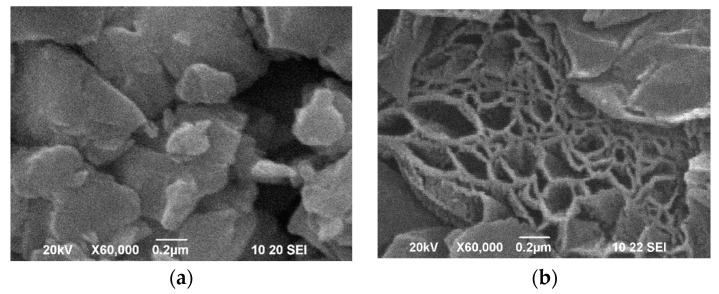

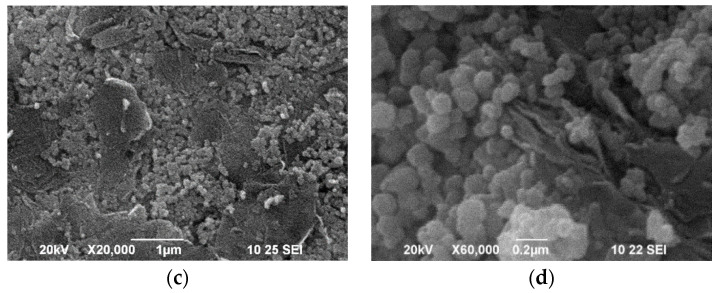
SEM images at higher magnifications for Ca_98_ExGr (**a**), Ca_98_NrGO (**b**), Zn_80_ExGr (**c**), and Zn_80_NrGO (**d**). These composites display the best figure of merit.

The common feature of composites of Ca_3_Co_4_O_9_ with ExGr and NrGO is the formation of a network comprising well-wrapped micrometric particles with flake-like particles. This is better manifested by the SEM images taken at a higher magnification (Figure 10): the oxide particles are well embedded into the NrGO’s thinner flakes, while for ExGr, the thicker flakes serve as a binder between oxide particles. The manner of particle packing ensures good contact between oxide and carbon particles, which is of importance when regulating the thermoelectric properties of composites. In addition, the intrinsic properties of ExGr and NrGO also play a significant role. The best thermoelectric activity of Ca_98_NrGO is mainly deu to the simultaneous decrease in the thermal conductivity and preservation of the Seebeck coefficient. The figure of merit of Ca_98_NrGO reaches a magnitude of 0.03 at room temperature, which is lower than the highest reported one for the Ba_0.27_CoO_2_ thin film (i.e., figure of merit of 0.11 along in-plane) [45]. In comparison with Ca_98_NrGO, Ca_98_ExGr adopts a slightly lower figure of merit (i.e., about 0.025) due to its bigger thermal conductivity. This is related to the different role of ExGr and NrGO regarding the the interface modification. The lower electrical resistivity of ExGr determines its bigger thermal conductivity compared to that of NrGO (Table 1).

**Figure 11:**
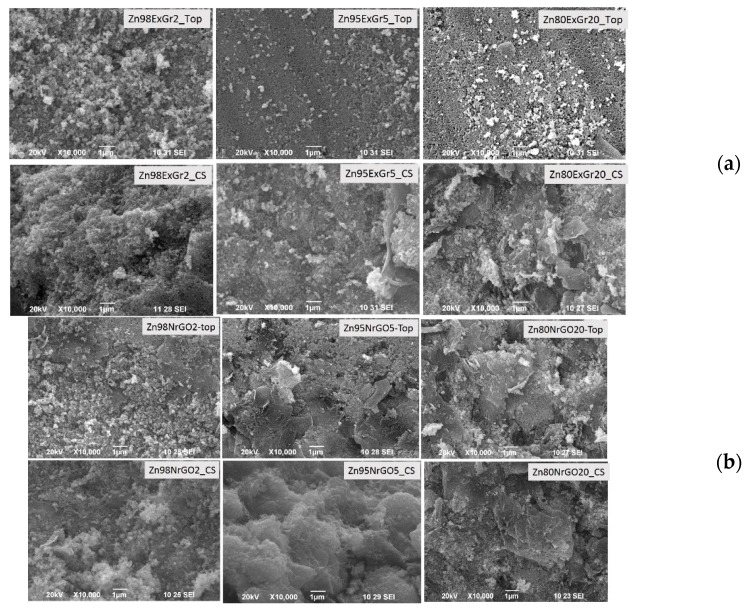
SEM images of the pellets (top and cross-sectional view) of Zn_0.995_Al_0.005_O-ExGr (**a**) and Zn_0.995_Al_0.005_O-NrGO composites (**b**). The carbon additives increase from 2 to 20 wt.% (from **left** to **right**).

The next peculiarity is observed when Ca_3_Co_4_O_9_ is replaced with Zn_0.995_Al_0.005_O (Figure 11). In this case, it appears that ExGr wraps better the nanometric oxide particles when the carbon content is 5 wt.%. In addition, the top of the pellet appears to be richer in oxide particles in comparison with the pellet’s cross-section. The addition of NrGO produces the same picture as is observed for ExGr additives: the oxide particles are well wrapped by NrGO at a content of 5 wt.% and they are more concentrated on the pellet top. It is noticeable that the morphological peculiarities of Zn_0.995_Al_0.005_O-based composites are opposite to those observed for Ca_3_Co_4_O_9_-based composites. This is confirmed by an EDS analysis of the C-to-Zn ratio on the top of and inside in the pellet (Figure 9): the top layers of the pellet become richer in oxide particles (expressed by amount of Zn). Irrespective of the different distribution of carbon additives along the pellets, it is important that, for both type of oxide composites, the carbon particles ensure a good contact between oxide particles, which is of importance for the modification of their thermoelectric properties (Figure 11).

## 4. Conclusions

Among the carbon-based materials, the expanded graphite (ExGr) exhibits the lowest electrical resistivity and negative Seebeck coefficient, thus converting it to an n-type thermoelectric material with an amazing power factor (PF), even at room temperature (71 μW/(m.K^2^) at 20 °C). Despite the relatively good magnitude of the power factor, the thermal conductivity of ExGr is high, which gives rise to its low figure of merit. Contrary to ExGr, both rGO and NrGO are characterized by extremely low thermal conductivities (more than two orders lower than that of ExGr), but their figures of merit remain small due to their high electrical resistivity and low Seebeck coefficient. A comparison of the thermoelectric properties shows that NrGO outperforms the rGO analogue, thus motivating the use of only NrGO as a component in multiphase composites.

Through ball-milling, composites of oxides and carbon additives are formed, where every individual component retains its structure. The common feature of composites is the formation of a network of well-wrapped oxide particles with flake-like carbon-based particles, thus ensuring good contact between oxide and carbon particles. After pelleting the Ca_3_Co_4_O_9_–carbon composites, the pellet top is enriched on carbon additives in comparison with the cross-sectional area. In the case of Zn_0.995_Al_0.005_O-carbon composites, the opposite trend is observed—the pellet top appears to be richer in oxide particles in comparison with the pellet’s cross-sectional area. The different manner of oxide packing is related to their morphology: for Ca_3_Co_4_O_9_, well-shaped micrometric particles dominate, while nanoparticles account for Zn_0.995_Al_0.005_O.

The manner of oxides’ particle packing and the intrinsic properties of ExGr and NrGO regulate the thermoelectric properties of composites. The thermoelectric activity of p-type Ca_3_Co_4_O_9_ is improved when 2 wt.% of ExGr or NrGO is added. The improved thermoelectric activity is a result of the simultaneous decrease in the thermal conductivity and preservation of the Seebeck coefficient. It is of importance that the thermal conductivity of the Ca_3_Co_4_O_9_-based composites decreases even in the case when individual components Ca_3_Co_4_O_9_ and ExGr exhibit high thermal conductivity. This is related to the enhanced phonon scattering in composites due to the heterostructural distribution of carbon–oxide phases. For the n-type Zn_0.995_Al_0.005_O, it is necessary to add more than 5 wt.% of carbon additives to enhance thermoelectric activity. Among ExGr and NrGO, only ExGr leads to a drastic augmentation of the figure of merit of Zn_0.995_Al_0.005_O (i.e., more than three orders of magnitude) due to a strong decrease in the electrical resistivity.

The established correlations between the thermoelectric properties of composites and their morphology and the amount of individual components could be used for further optimization of the thermoelectric performance of oxide materials at room temperature.

## Figures and Tables

**Table 1:** Electrical resistivity, Seebeck coefficient, power factor, thermal conductivity, and figure of merit for ExGr, rGO, NrGO, Ca_3_Co_4_O_9_, and Zn_0.995_Al_0.005_O, measured at 298 K.

Samples	ρ Ω.cm	S μV/K	PF = S^2^/ρ µW/(m·K^2^)	λ W/m.K	Figure of Merit S^2^T/(ρ·λ)
ExGr	9.6 × 10^−4^	−26.1	71	0.838	0.0255
NrGO	4.45 × 10^−2^	−2.8	1.7 × 10^−2^	0.003	0.0017
rGO	7.98 × 10^−2^	−0.6	3.9 × 10^−4^	0.005	2 × 10^−5^
Ca_3_Co_4_O_9_	3 × 10^−2^	94	29	0.620	0.0130
Zn_0.995_Al_0.005_O	4.6 × 10^3^	−590	8 × 10^−3^	0.300	8 × 10^−6^

## Data Availability

Data are contained within the article and Supplementary Materials.

## Supplementary Materials

The following supporting information can be downloaded at: https://www.mdpi.com/article/10.3390/ma16237262/s1, Figure S1: In-house setup for the measurement of the Seebeck coefficient; Figure S2: SEM-EDS analysis of the element content of Ca_95_ExGr_5_ composite.
