# Supplementary material for: Graphene-Based Composites for Thermoelectric Applications at Room Temperature
Source: Materials (Basel). 2023 Nov 21;16(23):7262. doi: 10.3390/ma16237262 (PMC10706976; doi:10.3390/ma16237262)
Supplement: Supplementary file 1 [file materials-16-07262-s001.zip › materials-2703917-supplementary.pdf]

Supplementary Materials

Article

Graphene-Based Composites for Thermoelectric Applications at Room Temperature

Sonya Harizanova [1,*], Vassil Vulchev [2] and Radostina Stoyanova [1,*]

[1] Institute of General and Inorganic Chemistry, Bulgarian Academy of Sciences, 1113 Sofia, Bulgaria
[2] Faculty of Physics, University of Sofia, 1164 Sofia, Bulgaria; vdv@phys.uni-sofia.bg
* Correspondence: sonya@svr.igic.bas.bg (S.H.); radstoy@svr.igic.bas.bg (R.S.)

The Seebeck coefficient of the samples has been measured at room temperature using the in-house setup. The holder comprises of a two solid plates with thermocouples. One of the plates contains gradient heater. The measured sample was inserted between the solid plates (Figure S1). Then the Seebeck voltage and temperature difference were recorded at reaching stationary conditions.

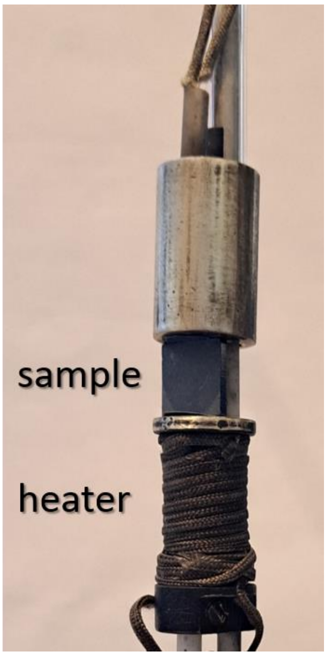

Figure S1. in-house setup for the measurement of the Seebeck coefficient.

The element content was determined from SEM-EDS analysis (Figure S2).

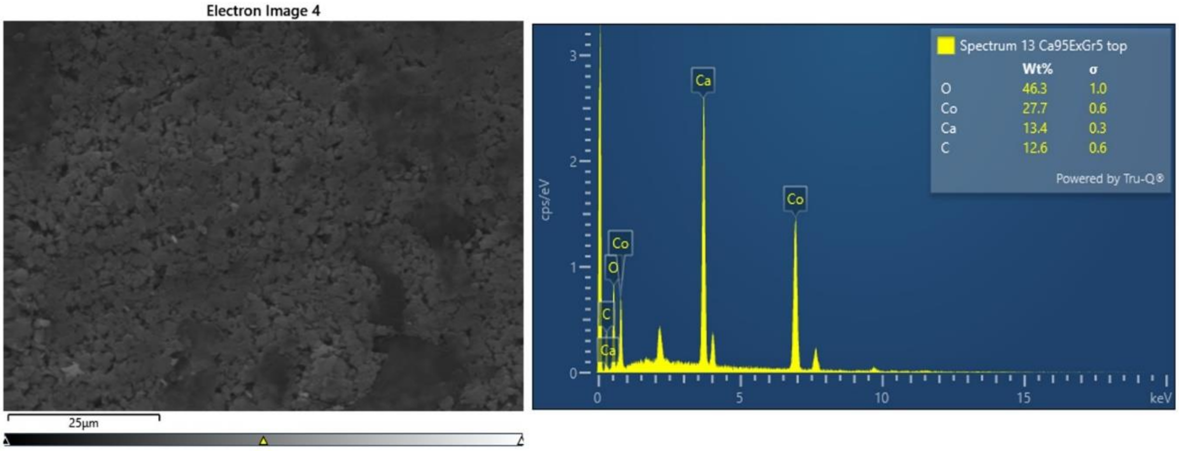

Figure S2. SEM-EDS analysis of the element content of Ca95ExGr5 composite.
